# NIR Electrofluorochromic Properties of Aza-Boron-dipyrromethene Dyes

**DOI:** 10.1038/srep18867

**Published:** 2016-01-06

**Authors:** Hanwhuy Lim, Seogjae Seo, Simon Pascal, Quentin Bellier, Stéphane Rigaut, Chihyun Park, Haijin Shin, Olivier Maury, Chantal Andraud, Eunkyoung Kim

**Affiliations:** 1 Department of Chemical and Biomolecular Engineering, Yonsei University, 50 Yonsei-ro, Seodaemun-gu, 120-749 Seoul, Republic of Korea; 2 CNRS-UMR 5182, Ecole Normale Supérieure de Lyon, Université de Lyon1, 46 Allée d’Italie, 69007 Lyon, France; 3 UMR 6226 CNRS-Université de Rennes 1, Institut des Sciences Chimiques de Rennes, 263 Av. du Général Leclerc, F-35042, Rennes Cedex, France

## Abstract

The photophysical properties of near-infrared (NIR) emissive aza-boron-dipyrromethene (aza-BDP) dyes incorporating nitrofluorene and alkoxy decorations were intensively investigated. Their highly reversible one-electron reduction process showed characteristic electrofluorochromic (EF) properties in the NIR range, depending on the substituents. The nitrofluorene ethynyl-substituted (Type I) dyes showed smaller EF effects than the alkoxy-containing (Type II) dyes because of the difference in their intrinsic fluorescence contrast between the neutral and reduced states (radical anion). In addition, the Type II chromophores showed a larger diffusion coefficient for ion transport, which enhanced the EF contrast and the response time for the fluorescence change at a given step potential. With an optimized condition, the NIR EF ON/OFF ratio reached a value of 6.1 and a long cyclability over 1000 EF cycles between −0.4 V and +0.4 V switching potentials, with approximately 20% loss of the initial ON/OFF switching ratio. The NIR EF switching was visually observed through a visible light cut-off filter, featuring high fluorescence contrast.

To date, stimuli-responsive fluorescence modulation has been the essential mechanism in ion sensing^1^,^2^, bio-analysis^3^,^4^, reversible control for optical memories^5^,^6^, and informational displays^7^,^8^,^9^,^10^,^11^,^12^. Specifically, fluorescence modulation based on the electrochemical reduction process, which is known as electrofluorochromic (EF) property, has been receiving more and more attention because it can provide a reversible fluorescence signal from an electrical stimulation^7^,^8^,^10^,^11^,^12^,^13^,^14^,^15^. This unique characteristic could be used as a fluorescence marker to determine the redox status of many active materials in bio-analysis^16^,^17^. Specifically for the bio-analysis application, the use of NIR-emissive EF materials could be a promising approach because it can provide non-invasive and background-signal-free images^18^. In this context, we recently reported the EF property of NIR-emissive polymethine dyes using their reversible fluorescence, switching between the NIR fluorescent state and the quenched state^9^,^19^. The main difficulties encountered in the development of the electrochemically active and NIR fluorescent materials are twofold: (i) insufficient fluorescence contrast and (ii) poor reproducibility. The ON/OFF ratio, which can be defined as the dimensionless value of ON state fluorescence intensity divided by OFF state, or fluorescence contrast, (~1.5) and the cyclability (~100 cycles) for the NIR EF switching of polymethine dyes are much smaller when compared with those of other EF switching materials in the visible region^9^. This low cyclability originates from the partially reversible one-electron oxidation process of the chromophores. Because EF is driven by the electroconversion degree of the electroactive EF material, the chemical reversibility of the redox event (or the stability of the various redox forms) should be primarily considered to achieve a high EF contrast. Among the various electroactive materials^20^,^21^,^22^,^23^,^24^,^25^, aza-boron-dipyrromethene (aza-BDP) dye can be a promising candidate because of its reversible electrochemical properties and various structural availability with different substituents^26^,^27^. It is well known that the electrochemical behaviors of analogous carbon-BDP dyes are strongly dependent on the substituents, which may prevent the decomposition reaction of the generated radical ions^22^,^23^,^24^. Furthermore, aza-BDP dyes generally possess an intense fluorescence emission band centered approximately 500–700 nm and are shifted to a lower energy when compared with carbon-BDP. Several studies have shown that the photophysical properties of aza-BDPs can be further red-shifted to the NIR region by the functionalization with an electron-donating group^28^,^29^,^30^ and the extension or planarization of the conjugated pathway^31^,^32^,^33^,^34^. In this context, several of us recently described aza-BDPs substituted by nitrofluorene ethynyl moieties at the 1/7 or 3/5 positions, **1** and **2,** respectively, that present intense emission in the NIR spectral range (Fig. 1)^35^. Although the nitro group may not be a common substituent for fluorescent materials, because of their quenching possibility, the fluorescence quenching via charge transfer of the **1**, **2** and **3** dye were hardly observed. Thus, the nitro group seems appropriate as an electron-withdrawing groups for aza-BDPs. Here, we describe the synthesis of **3** functionalized in the 2/6 position and compare the photophysical and electrochemical properties with those of chromophores **4** and **5**, featuring alkoxy donor substitution^36^. Finally, we report the highly reversible electrochemical NIR fluorescence switching of these aza-BDPs featuring either Type I or Type II substituents. Ultimately, the high contrast and highly reversible NIR EF switching were achieved by the precise control of the applied potentials.

## Experimental

### Materials and methods

Silver wire (d = 0.1 mm) was purchased from The Nilaco Corporation in Tokyo, Japan and used as the reference electrode in a three-electrode device. Butvar B-98, Tetrabutylammonium hexafluorophosphate (TBAPF_6_), dichloromethane (MC), and dimethyl sulfoxide (DMSO) were purchased from Sigma-Aldrich. ITO glass was cleaned according to the reported procedures^37^. NMR spectra (^1^H, ^13^C) of all the new molecules were recorded at room temperature on a BRUKER Avance operating at 500.10 MHz and 125.75 MHz for ^1^H and ^13^C, respectively and on a BRUKER Avance operating at 188.81 MHz for the ^19^F experiments. Chemical shifts are listed in parts per million (δ, ppm) and are reported relative to the residual solvent peaks used as an internal standard (For ^1^H and ^13^C, respectively: CDCl_3_: 7.26 and 77.2 ppm; CD_2_Cl_2_: 5.32 and 53.8 ppm). High-resolution mass spectrometry measurements were performed at the “Centre Commun de Spectrometrie de Masse” (Villeurbanne, France). Absorption spectra were recorded on a JASCO V-650 spectrophotometer in a diluted solution (*ca.* 10^−5^ or 10^−6^ mol.L^−1^) using spectrophotometric grade solvents. Molar extinction coefficients (ε) were precisely determined at least two times. Emission spectra were measured using a Horiba-Jobin-Yvon Fluorolog-3 iHR320 fluorimeter. Short luminescence decay was monitored with the TC-SPC Horiba apparatus using Ludox in distilled water to determine the instrumental response function needed for deconvolution. Excitation was performed using NanoLEDs, with models (peak wavelength; pulse duration) 570 (573 nm; 1.5 ns) and 740 (732 nm; 1.3 ns). The deconvolution was performed using the DAS6 fluorescence-decay analysis software. Luminescence quantum yields *Q* were measured in diluted solutions with an absorbance lower than 0.1 using the following equation: *Q*_x_/*Q*_r_ = [*A*_r_(λ)/*A*_x_(λ)][n_x_^2^/n_r_^2^][*D*_x_/*D*_r_], where *A* is the absorbance at the excitation wavelength (λ), n the refractive index, and *D* is the integrated luminescence intensity. The subscripts “r” and “x” stand for reference and sample, respectively. Excitation of the reference and dyes was performed at the same wavelength.

### Synthesis

aza-BDP dyes **1**, **2**, **4,** and **5** were prepared following previously reported methods^35^,^38^. The synthesis of dye **3** is described in Scheme S1. Detail description of synthesis was informed in supporting section 1.

### Preparation of the three electrode EF devices

The three electrode EF switching device was consist of an electrolyte layer that is sandwiched between two ITO electrodes (13 Ω sq^−1^), and Ag wire (d = 0.1 mm) as a reference electrode. The polymer electrolyte was prepared by dissolving aza-BDP dyes (0.01 M) and TBAPF_6_ (0.2 M) in 20 ml of DMSO with 2.23 g of Butvar B-98 (10 wt %) as the host polymer^37^. Host polymer was added to make a gel type electrolyte which has high viscosity. In the highly viscous solution, the amount of TBAPF_6_ salt was doubled to compensate the low ion mobility in the gel type electrolyte. Before preparing the EF device, a 2 × 3 cm^2^ ITO glass (working) and drilled ITO glass (counter) were sonicated for 10 min in ethanol and acetone, and then dried at 100 °C. After drying the ITO glass, imide tape was attached as a spacer for the electrolyte layer. The switching device was prepared by assembling the ITO electrode and inserting Ag wire as a reference electrode between the working and counter electrodes. The electrolyte with host polymer was carefully injected through the hole of the counter electrode, and the hole was completely blocked with an epoxy resin after electrolyte filling the gap between the electrodes.

### Characterization of the EF devices

Electrochemical properties for the three EF devices in this study were examined using a universal potentiostat [model CHI 624B (CH Instruments, Inc.)]. It helps to calculate the injected/ejected charge during EF switching experiment. The graph which have time x axis and charge y axis showed delta charged value into EF devices. As a result, R% value in Table S1 was calculated. Cyclic voltammetry (CV) was performed after 5 min of nitrogen purging. Fluorescence spectra were obtained using a Model LS55 luminescence spectrometer (PerkinElmer). When recording the fluorescence along with the external voltage, *in situ* fluorescence of the switching device was obtained using a luminescence spectrometer. NIR photography was obtained with a digital camera (IR cut-off filter removed, 5D Mark III, Canon) equipped with a visible-light cut-off filter (720 nm cut-off filter) and with a 684 nm excitation source as shown in Fig. S1.

## Result and Discussion

**Figure 2:**
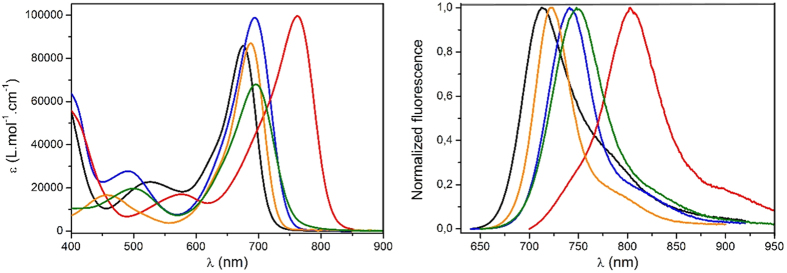
Absorption (left) and emission (right) spectra of dyes 1 (black), 2 (blue), 3 (red), 4 (orange) and 5 (green) in a dichloromethane solution.

**Figure 3:**
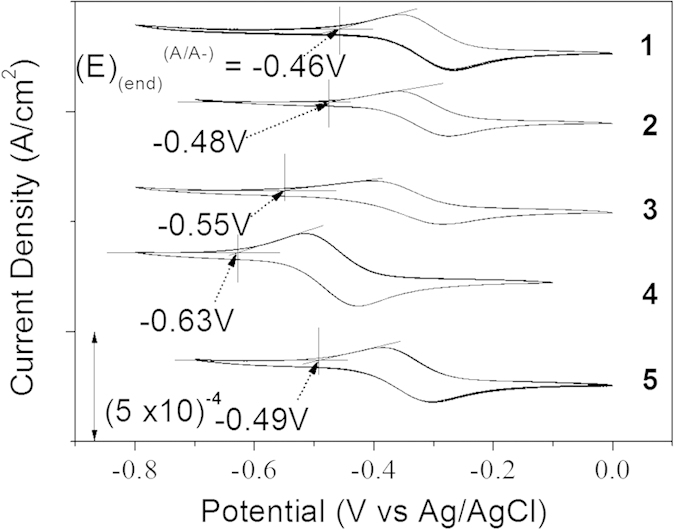
Cyclic voltammograms of NIR aza-boron-dipyrromethenes in an electrolyte containing TBAPF_6_ 0.2 M as a salt in dichloromethane (v = 100 mV/s). The dyes were dissolved in the electrolyte (10^−3^ M) and measured with a Ag/AgCl reference electrode, stainless steel counter electrode, and Pt disk working electrode.

**Figure 4:**
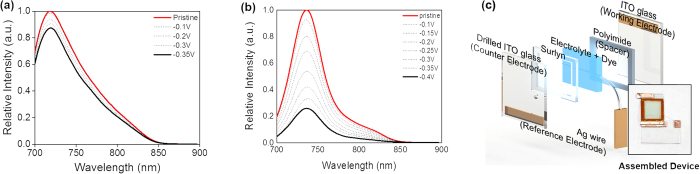
(**a,b**) represent the fluorescence changes of dyes **1** and **4** from neutral to reduced states respectively. The aza-boron-dipyromethene dyes were dissolved in DMSO solvent with Butvar B-98 polymers and biased with applied potentials ranging from neutral state (top) to their intrinsic reduction potential (bottom). Each spectrum was obtained after applying a target potential for 80 s. (**c**) Schematic image of the electrofluorochromic device consisting of ITO electrodes, a spacer, and an electrolyte layer. The inset shows images of the assembled device.

**Figure 5:**
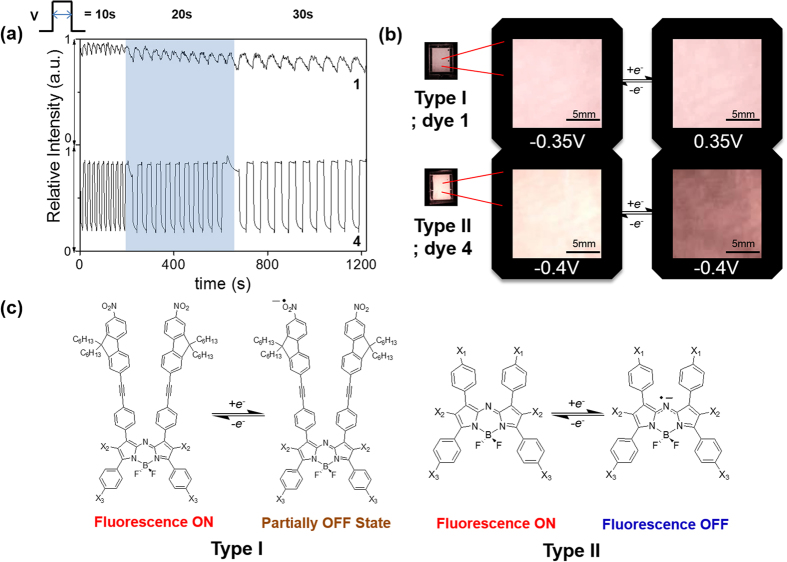
Electrofluorochromic switching responses of the aza-boron-dipyrromethene dyes with various step-duration time of 10 s, 20 s, and 30 s at each potential. The switching potential was applied from redox to neutral potential to compare the electrofluorochromic switching properties. The emission intensity was monitored at their emission maximum. (**b**) NIR images of the electrofluorochromic switching devices applied at their switching potential to show ON/OFF NIR emission switching. The ON/OFF fluorescence images were captured with <720-nm cut-off filters under a diffused excitation light (λ_max_ = 684 nm). (**c**) The possible mechanism for the electrofluorochromic switching observed in Type I and Type II dyes.

The new aza-BDP **3** featuring nitrofluorene ethynyl substituents at positions 2/6 was easily obtained by Sonogashira cross-coupling between a dihalogenated aza-dipyrromethene (Scheme S1) and the nitrofluorene ethynyl synthon^39^,^40^,^41^. Further chelation to boron trifluoride etherate in a basic medium afforded dye **3** to have a good yield. This dye and the intermediates were characterized by ^1^H and ^13^C NMR and HRMS (see supporting information). The absorption and fluorescence properties of chromophores **1–5** were recorded in a diluted dichloromethane solution (ca. 10^−5^ M). The spectra are presented in Fig. 2, and the photophysical data are compiled in Table 1. The photophysical properties of aza-BDPs were also dependent on the electronic and steric nature of substituents. The aza-BDP dyes functionalized at the 1/7 and 3/5 positions display intense and sharp absorption transitions that are centered approximately at 690 nm. The introduction of the nitrofluorene ethynyl functions at the 2/6 positions in dye **3** leads to a dramatic red-shift of the absorption towards ~760 nm. The emission spectra of dyes were also influenced significantly by the substituents. Indeed, while dye **1** with substituents at the 1/7 positions shows a moderate fluorescence quantum yield at 675 nm (Φ = 13%), the emissions of the aza-BDPs functionalized at the 3/5 positions (**2**, **4**, **5**) were more intense (Φ = 24–36%) and slightly red-shifted, particularly in the cases of **2** and **5,** featuring an extended conjugation. Notably, **1** exhibits a lower quantum yield and fast lifetime when compared with **2** possibly because the π-conjugated substituents at 3/5 positions can prevent free rotation and, hence, increase non-radiative deactivation processes^35^. The rigidification of aza-BDP dyes could result in the red-shifted, intense emission profile using the intramolecular interaction, such as hydrogen bonding^42^ or π–π interaction^43^. The dyes in this work were designed to induce the rigidification with planar aza-BDP core. This planar structures were confirmed by the calculation using the DFT method (Fig. S2) with the average value between the phenyl group and the aza-BDP core. Interestingly the emission property of the aza-BDPs was well matched to the planarity of the dye molecules (Fig. S3): Planarity of **2** is slightly higher than the analogue structure of **1**, so the fluorescence band of **2** is observed at more red-shifted region with a high quantum yield. On the other hand, the fluorescence quantum yield of **4** and **5** are similar because the planarity of two dyes were quite similar. Functionalization at the 2/6 positions in dye **3** resulted in a red-shift of the emission, with a maximum located in the NIR range at 803 nm. However, this significant shift was accompanied by a severe decrease in the fluorescence quantum yield and lifetime. This fluorescence quantum yield decrease in dye **3** cannot be ascribed to the substitution at the 2/6 positions, but to the reduced energy band gap and their distorted structure. Indeed, it is well known that the red-shift emission up to the NIR region is generally accompanied by a strong decrease of the quantum yield possibly due to a favorable non-radiative decay caused by the reduced energy gap in NIR region^35^. Also, as shown in Figs S2 and S3, the distorted structure of **3** may affect to decrease the quantum yield. As a result, the introduction of electron-donating groups or elongation of the conjugation led a red-shift in both absorption and emission, which enabled the formation of NIR emissive aza-BDPs. The red-shift emission at the NIR region was unfortunately accompanied by a strong decrease of the luminescence, as shown in dye **3**. To use the aza-BDP dyes for the elaboration of a reversible EF device, the electrochemical properties were examined by cyclic voltammetry (CV). The one-electron reduction and oxidation waves of the aza-BDPs are shown in Figs 3 and S4, respectively. The stability of the radical anion and cation forms after respective reduction and oxidation of the BDP are known to be strongly dependent upon the substituents^23^. Specifically, the absence of a substituent on position 8 could lead to decomposition by dehydrogenation or by nucleophilic substitution reactions, resulting in irreversible electrochemical reactions^44^. However, these decomposition reactions are intrinsically prevented by replacing carbon with nitrogen in the aza-BDP^23^,^24^,^25^. Therefore, the one-electron reduction process is highly reversible because the decomposition pathway from position 8 of the aza-BDPs is completely prevented. Interestingly, the E_1/2,A/A-_ values of **1, 2,** and **3** were comparable, irrespective of the position of the nitrofluorene ethynyl groups. Overall, the substitution position of the Type I do not have a notable effect on the reduction potential. Although the methoxy group was substituted in dye **3**, it appears that the nitrofluorene ethynyl groups prevented the potential shift, which is observed in E_1/2,A/A-_ of **4**. Thus, the value for **3** was positioned near **1** and **2**. Compared with **4**, the E_1/2,A/A-_ of dye **5** has fewer negative values, possibly because the methoxy group does not directly substitute onto the aza-BDPs center. However, the reversibility of one-electron oxidation process is partially dependent on the substituents, because of the instability of the resulting cation radical. The radical cations of the aza-BDPs are easily decomposed when there is no substituent in the C2, C3, C5, or C6 positions^44^. Thus, the absence of a substituent on C2 and C6 in the aza-BDPs can result in dimerization or deprotonation. This is supported by the fact that **3,** for which the C2 and C6 positions are blocked by the substituent, presents a highly reversible oxidation process when compared with other dyes. It is noteworthy that dyes **1** and **2,** which are substituted by the bulky group, show a reversible oxidation process because the steric hindrance of the bulky substituent can prevent the decomposition. However, dyes **4** and **5** showed irreversible oxidation reaction, caused by the formation of unstable cation radicals^23^,^44^. Moreover, the fluorescence changes with the one-electron oxidation process was too small (Fig. S5) to use it as an EF switching. As EF switching occurs between the neutral and reduced forms, the reversible one-electron reduction process (Fig. 4) was selected for use in EF switching to obtain high-contrast switching without electrochemical decomposition. The working potential for EF switching based on the reduction process was determined electrochemically as shown in Figs 3 and S6. The CV showed highly stable reduction process within 0 V to −0.5 V. On the other hand, dyes were very unstable in oxidation potential higher than 1 V as shown in Fig. S4. It was noteworthy that aza-BDPs showed high electrochemical stability during EF switching based on the reduction process, because the nitrogen substituted aza-BDPs are resistant to the electrochemical decomposition. The EF device was prepared as shown in Fig. 4(c), and the solution containing the electrolyte and the dye was injected into device^20^. NIR emission of the device was determined upon applying a potential between the reduction potential and a reverse positive potential (against the Ag wire reference electrode) to minimize the decomposition. As shown in Fig. 4, the fluorescence emission of the aza-BDPs was quenched according to the applied potentials (E_1/2,A/A-_). Interestingly, this emission intensity at the quenched state was highly dependent on the chemical structure of the aza-BDPs. Type I showed low EF-switching contrast when compared with Type II. This could be attributed to the difference in the quenching efficiency (Ф_q_ = (A_r_/A_n_)*100), where A_r_ and A_n_ are the integrated area of the emission band at the reduced state and the neutral state, respectively. Because the absorbance changes according to the applied potentials were negligible in aza-BDPs (Fig. S7), the quenching efficiency may represent the quantum yield decreases. Based on the fluorescence spectra in Figs 4 and S8, the Ф_q_ of **1** and **2** was determined as 86.4% and 76.3%, respectively. Whereas, that of **3**, **4**, and **5** was determined as 40.2%, 25.5%, and 55.2%, respectively, under the application of their E_1/2,A/A-_. These values could be slightly shifted by the uncontrolled experimental condition with ~20% error range, but each aza-BDP dyes showed stable EF properties under the intrinsic electrochemical conversion. A possible explanation for this difference follows. Considering the electrochemistry of a typical aza-BDP^35^, the radical generated from the one-electron reduction process could be positioned at position 8, resulting in fluorescence quenching. However, Type I has an electron-deficient group, at which the electron could be delocalized during the reduction process^45^. Therefore, the fluorescence of the aza-BDPs structure can remain even after the application of E_end_,_A/A-_. However, the one-electron reduction process of the Type II dyes is not affected by the substituents, as they are electron-donating groups, resulting in sufficient fluorescence contrast. The Ф_q_ of **5** was lower than **4** possibly because of the presence of a conjugation extension in **5**. Interestingly, the Ф_q_ of **3** was ranked second, which is higher than that of **5**. It might be affected by the strong effect of the methoxy group rather than the side chain of **5** in spite of the nitrofluorene ethynyl substituent. Because the radical is possibly generated at N8, which is in the central ring of the aza-BDPs, the decrease in the fluorescence quantum yield would be sufficient to elucidate the ON/OFF switching. Additionally, when the applied potential was increased to the end potential for reduction, the EF contrast differences between Type I and Type II were also observed in the EF switching by the step potential application between the neutral and reduced potential. The NIR EF switching devices were operated with varying step-duration times from 10 s to 30 s, as shown in Fig. 5. Based on the applied potential duration, the fluorescence emission contrasts became larger when the duration times at each potential step were longer. The Type II dyes showed higher switching contrast than the Type I dyes. The maximum ON/OFF ratio, determined by dividing the ON state emission intensity with the OFF state intensity, was 2.4, 6.1, and 1.6, respectively, for dyes **3**, **4,** and **5**, with a 300 s duration time. Specifically, this ON/OFF switching of **4** was reversibly continued over 1000 cycles with a 10 s duration time with less than ~20% loss of the initial ON/OFF ratio (Fig. S9). However, the ON/OFF ratio of **1** and **2** was not as high as that for the Type II dyes because of a previously stated reason. In the case of **3,** it could be categorized as Type I but also II because it contains both nitrofluorene ethynyl (Type I) and alkoxy group (Type II), at 2/6 and 3/5 position, respectively. The ON/OFF ratio slowly decreased after repeated switching cycles. This may be ascribed to the electrochemical decomposition, such as dimerization and deprotonation. Especially, the unavoidable electrochemical oxidation can be occured on the counter electrode under the applcaition of the negative potentials at the working electrode. Because the oxidation process can result in the electrochemical dimerization, the electrochemical decomposition in a repeated EF switching can be observed with a slight decrease of ON/OFF ratio. Interestingly, the effect of the alkoxy group on the EF switching for **3** was greater than that of the nitrofluorene ethynyl. Therefore its property became similar to Type II dyes as shown in Fig. S10. Furthermore, the ON/OFF ratio for 300 s step time of **3** was larger than **5** and was similar to that of dye **4**. This could be attributed to the methoxy group of **3** at 3/5 position, while **5** contains a longer alkoxy and bridging group that may deter the EF switching. Similarly, the response time for **4** and **5** was much faster than **1** and **2**, and that for **3** was faster than **2** (Fig. S10). This indicates that the EF response is highly affected by the electronic nature of the substituents and the dye substituted methoxy group directly at 3/5 position showed the best EF properties. Thus **4** showed the highest ON/OFF ratio with the fastest response, while those EF properties become worse in a dye with a longer alkoxy group substitution (**5**). Furthermore the EF properties become worst when nitrofluorene ethynyl groups are substituted on a dye (Fig. S10 and S11(a)). Thus **1** showed the worst EF response while **3** was in-between, owing to the methoxy and nitrofluorene ethynyl groups. Moreover, the ON/OFF ratios of dye **1** and **2** were still less than for Type II. This relatively low switching contrast was not only caused by the intrinsic fluorescence contrast of the dye but also by the injected/ejected charge with 10 s and 50 s duration time in DMSO and MC solutions as shown in Table S1 and S2. Because this ON/OFF switching is based on the reduction process, the amount of injected/ejected charge during switching should be quantitatively related to the reduction of the dye. As described in the supporting information, the amount of the reduced dye was significantly different, varying from 0.3 to 40.5%. For the highest contrast dye **4**, the injected/ejected charge was also much higher than for the others, which was possibly because of the high diffusion coefficient^22^,^24^,^46^. According to the Randles-Sevcik equation, the anodic and cathodic peak current for the one-electron reduction process was linearly related to the square root of the scan rate, and the diffusion coefficient was calculated from the slope^47^. As shown in Supplementary Fig. S12, dye **4** showed the highest diffusion coefficient of 1.5 × 10^−10^ cm^2^/s in MC solvent. The fast kinetics of the dye can result in a fast electrochemical response, accompanied by the enhanced ON/OFF contrast. However, the remaining Type II dyes, **3** and **5,** showed moderate switching contrast during EF switching, possibly because of the rather slow diffusion, with each having a diffusion coefficient of 4.0 × 10^−11^ cm^2^/s and 4.3 × 10^−11^ cm^2^/s, respectively. The injected/ejected charge during the switching was not as high as for **4**; therefore, **3** and **5** exhibited lower switching contrasts than **4**. As shown in Table S1, the amount of injected/ejected charge for **4** was higher than the others. This results can be rationalized by the high diffusion coefficient of the dye. Additionally, the fast diffusion was essential to achieve the fast EF response. In Figs 5 and S13, the EF switching response of **4** was faster than the others and shows a square-type EF response, which indicates the electroconversion is saturated during these times.

After investigating the EF properties, EF switching was visually achieved through the optical filter. Upon exposure to a laser light source (684 nm), the switching device showed fluorescence in the NIR range. This NIR emission passed through an optical filter to remove background light and was thus photographed by a digital camera. The images in Fig. 5(b) show the NIR switching that results from the device containing the aza-BDP dyes. The neutral state intensity represents the fluorescence quantum yield of the dye, because the images were obtained in the same experimental condition. Therefore, the observed NIR intensity from chromophore **3** was negligible when compared with the other device because of a very low quantum yield (<1%). The Type I dyes showed slight EF switching upon the applied potential, but the switching contrast was not as high as for the Type II dyes. The possible electrochemical conversion during the one-electron reduction process is shown in Fig. 5(c). The fluorescence quenched radical anion state of Type II enabled high-contrast EF switching. Specifically, **4** showed the highest contrast EF switching among all the dyes examined thus far. This is possibly because of the effective reduction process for the EF switching, as implied by the high diffusion coefficient of **4**. This NIR switching was observed visually, as shown by the digital camera image and movie. (Supporting movie).

## Conclusion

This paper demonstrates highly reversible EF switching with NIR emissive aza-BDP dyes. The NIR emission was achieved using two different series of aza-BDP dyes, incorporating nitrofluorene ethynyl (Type I) and alkoxy (Type II) functionalization at 3/5, 1/7, or 2/6 positions. Both types of chromophore exhibited NIR emission between 700 nm and 800 nm with a moderate quantum yield. Interestingly, both types of dyes showed a reversible one-electron reduction process; however, the EF properties were significantly different. Because of the electron-withdrawing nitro group on the Type I dyes, the fluorescence quenching of the radical anion state was not fully quenched. However, the Type II dyes exhibited a higher switching contrast because of the absence of an electron-withdrawing group. Specifically, the fast diffusion of **4** enabled a high EF contrast and response time. The device containing dye **4** showed the highest contrast (6.1 of ON/OFF ratio) and longest switching cycles (>1000, at +0.4 V ~20% loss of the initial switching ON/OFF ratio.) among the NIR EF switching materials studied thus far.

## Additional Information

**How to cite this article**: Lim, H. *et al.* NIR Electrofluorochromic Properties of aza-Boron-dipyrromethene Dyes. *Sci. Rep.* **6**, 18867; doi: 10.1038/srep18867 (2016).

## Supplementary Material

Supplementary Movie S1

Supplementary Movie S2

Supporting Information

## Figures and Tables

**Figure 1:**
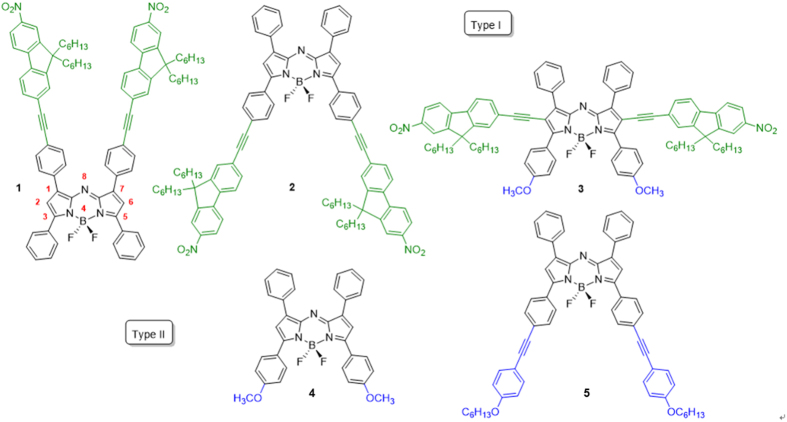
Structure of aza-boron-dipyrromethene dyes studied in this work. The red numbers represent substituent positions.

**Table 1:** Photophysical and electrochemical properties of aza-boron-dipyrromethene dyes in dichloromethane.

Dyes	λ_abs_ (nm)/ε (10^3^.L.mol^−1^.cm^−1^)	λ_em_(nm)	Φ (%)	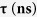	E_1/2_ (V vs Ag/AgCl)	
					A/A^−^	A/A^+^
**1**	675/86	711	13^a^	1.5	−0.31	1.33
**2**	694/100	741	36^a^	2.7	−0.32	1.28
**3**	762/100	803	1^b^	<0.4	−0.34	1.15
**4**	687/87	721	28^c^	–	−0.47	1.37^d^
**5**	696/68	748	24^c^	2.5	−0.34	1.19

^a^From ref^35^.

^b^IR-125 as reference (DMSO, Φ = 13%).

^c^Cresyl Violet as reference (MeOH, Φ = 55%).

^d^Oxidation of dye **4** is partially reversible.
