# Supplementary material for: NIR Electrofluorochromic Properties of Aza-Boron-dipyrromethene Dyes
Source: Sci Rep. 2016 Jan 6;6:18867. doi: 10.1038/srep18867 (PMC4702066; doi:10.1038/srep18867)
Supplement: Supporting Information [file srep18867-s3.doc]

**Supplementary Information**

**NIR Electrofluorochromic Properties of Aza-Boron-dipyrromethene Dyes**

Hanwhuy Lim,^1^ Seogjae Seo,^1^ Simon Pascal,^2^ Quentin Bellier,^2^ Stéphane Rigaut,^3^ Chihyun Park,^1^ Haijin Shin,^1^ Olivier Maury,^2^ Chantal Andraud^2^* and Eunkyoung Kim^1^*

*1 Department of Chemical and Biomolecular Engineering, Yonsei University, 50 Yonsei-ro, Seodaemun-gu, 120-749 Seoul, Republic of Korea. E-mail:* [*eunkim@yonsei.ac.kr**](mailto:eunkim@yonsei.ac.kr*)

*2 CNRS-UMR 5182, Ecole Normale Supérieure de Lyon, Université de Lyon1, 46 Allée d’Italie, 69007 Lyon, France. E-mail:* [*chantal.andraud@ens-lyon.fr**](mailto:chantal.andraud@ens-lyon.fr*)

*3 UMR 6226 CNRS-Université de Rennes 1, Institut des Sciences Chimiques de Rennes, 263 Av. du Général Leclerc, F-35042, Rennes Cedex, France*

**1. Preparation of aza-boron-dipyrromethene dye 3**

**
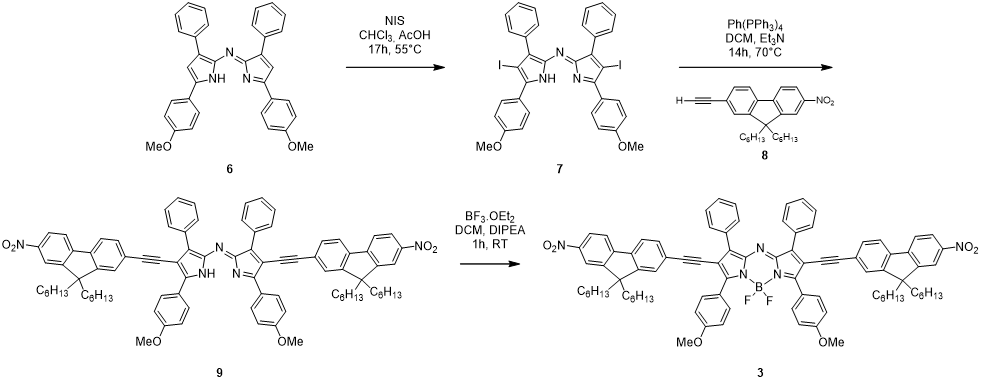
**

**Scheme S1.** Synthesis steps of aza-boron-dipyrromethene dye **3**.

Compound **7**. A round-bottom flask was charged with a solution of 200 mg of **6** (0.39 mmol, 1 eq.) in 20 mL of degassed CHCl_3_ and acetic acid glacial (3:1). Next, 194 mg of *N*-iodosuccinimide (0.86 mmol, 2.2 eq.) was added and the reaction was stirred for 17 hours at 55 °C under an Argon atmosphere. The crude reaction mixture was diluted in dichloromethane and washed with a Na_2_O_5_S-saturated aqueous solution (30 mL), a Na_2_CO_3_-saturated aqueous solution (30 mL), and water (30 mL). The aqueous layers were extracted with dichloromethane (2 x 10 mL), and the combined organic layers were dried over Na_2_CO_3_ and concentrated. The crude residue was purified by dissolution in a minimum of CH_2_Cl_2_ and precipitation by the addition of pentane. The product was obtained as a dark blue solid with 87 % yield (260 mg). ^1^H NMR (CD_2_Cl_2_, 500.10 MHz): δ 8.00 (d, ^3^J = 8 Hz, 4H, CH_Ar_), 7.68 (d, ^3^J = 6.4 Hz, 4H, CH_Ar_), 7.42-7.34 (m, 6H, CH_Ar_), 7.05 (d, ^3^J = 8 Hz, 4H, CH_Ar_), 3.90 (s, 6H, OCH_3_). ^13^C NMR (CD_2_Cl_2_, 125.75 MHz): compound was too insoluble to record spectra. MS (ESI+): [MH]^+^ = 762.0105 (calcd. for C_34_H_26_I_2_N_3_O_2_: 762.0109). UV-Vis (CH_2_Cl_2_) λ_max_ = 595 nm (ε_max_ = 36000 L.mol^-1^.cm^-1^).

Compound **7** appeared to be poorly soluble in polar solvents and no ^13^C NMR could be recorded in CD_2_Cl_2_. In order to fully characterize this intermediate, the boron complex of **7** was prepared. Thanks to an increase of the solubility, the characterization of aza-BDP **7‑BF_2_** could be completed by ^13^C NMR.

Compound **7-BF_2_**. Compound **7** (61 mg, 0.08 mmol, 1 eq.) was dissolved in 20 mL of CH_2_Cl_2_ purified by distillation and 0.13 mL of distilled DIPEA (0.8 mmol, 10 eq.) was added to the solution. The mixture was stirred for 1 h at RT. Then 0.13 mL of boron trifluoride etherate (1.05 mmol, 15 eq.) was added by syringe. The green reaction mixture was stirred overnight at RT and quenched with H_2_O (20 mL). After separation, the organic layer was dried over Na_2_SO_4_, filtered, and concentrated. The crude product was purified by flash chromatography on silica gel using petroleum ether/CH_2_Cl_2_ as eluent (1:1, R_f_ = 0.23). The product **7-BF_2_** was obtained as a dark blue solid in 56 % yield (36 mg). ^1^H NMR (CDCl_3_, 500.10 MHz): δ 7.78 (m, 4H, CH_Ar_), 7.69 (d, 4H, ^3^J = 9 Hz, CH_Ar_), 7.42 (m, 6H, CH_Ar_), 6.98 (d, 4H, ^3^J = 9 Hz, CH_Ar_), 3.86 (s, 3H, OCH_3_). ^13^C NMR (CDCl_3_, 125.75 MHz): δ 161.7 (C_quat_), 160.6 (C_quat_), 148.1 (C_quat_), 145.2 (C_quat_), 132.6 (CH), 132.1 (C_quat_), 130.9 (CH), 129.6 (CH), 128.0 (CH), 123.5 (C_quat_), 114.6 (C_quat_), 113.6 (CH), 55.4 (OCH_3_). ^19^F NMR (CDCl_3_, 188 MHz): δ -132.3 (q, J_F-B_ = 30 Hz). UV-Vis (CH_2_Cl_2_): λ_max_ = 677 nm (ε_max_ = 81000 L.mol^-1^.cm^-1^). HRMS (ESI^+^): [M+H]^+^ = 810.0096 (calcd for C_34_H_25_BF_2_I_2_N_3_O_2_: 810.0098).

Compound **9**. 200 mg of **7** (0.26 mmol, 1 eq.) and 274 mg of nitrofluorene acetylide derivative **8** (0.66 mmol, 2.5 eq.) were dissolved in 20 mL of degassed CH_2_Cl_2_ and Et_3_N (1:1), and the solution was added to 91 mg of Pd(PPh_3_)_4_ (0.08 mmol, 3 mol. %). The mixture was stirred for 14 h at 70°C and then allowed to cool to RT. The solution was diluted in CH_2_Cl_2_ (30 mL) and washed with a saturated aqueous solution of NH_4_Cl (30 mL) and brine (30 mL). The organic layer was dried over MgSO_4_, and after purification by flash chromatography on silica gel using CH_2_Cl_2_/petroleum ether as the eluent (5:5, R_f_ = 0.40), the product was isolated as a dark green solid with 69 % yield (239 mg). ^1^H NMR (CDCl_3_, 500.10 MHz): δ 8.36 (d, ^3^J = 8 Hz, 4H, CH_Ar_), 8.28 (dd, ^3^J = 8 Hz, ^4^J = 2 Hz, 2H, CH_Ar_), 8.24 (d, ^3^J = 8 Hz, 4H, CH_Ar_), 8.21 (d, ^4^J = 2 Hz, 4H, CH_Ar_), 7.79 (d, ^3^J = 8 Hz, 2H, CH_Ar_), 7.77 (d, ^3^J = 8 Hz, 2H, CH_Ar_), 7.50-7.43 (m, 10H, CH_Ar_), 7.11 (d, ^3^J = 8 Hz, 4H, CH_Ar_), 3.96 (s, 6H, OCH_3_), 2.03 (m, 8H, CH_2_), 1.15-1.03 (m, 24H, CH_2_), 0.77 (t, ^3^J = 7 Hz, 12H, CH_3_), 0.62 (m, 8H, CH_2_). ^13^C NMR (CDCl_3_, 125.75 MHz): δ 161.8 (C_quat_), 155.1 (C_quat_), 152.6 (C_quat_), 152.4 (C_quat_), 149.1 (C_quat_), 147.5 (C_quat_), 146.9 (C_quat_), 143.3 (C_quat_), 138.9 (C_quat_), 132.9 (C_quat_), 130.7 (CH), 130.7 (CH), 130.0 (CH), 128.5 (CH), 127.9 (CH), 125.8 (CH), 125.0 (C_quat_), 124.5 (C_quat_), 123.6 (CH), 121.4 (CH), 120.3 (CH), 118.4 (CH), 114.6 (CH), 111.0 (C_quat_), 98.5 (C_quat_), 87.8 (C_quat_), 55.9 (C_quat_), 55.7 (OCH_3_), 40.2 (CH_2_), 31.6 (CH_2_), 29.7 (CH_2_), 23.9 (CH_2_), 22.7 (CH_2_), 14.1 (CH_3_). UV-Vis (CH_2_Cl_2_): λ_max_ = 656 nm (ε_max_ = 62500 L.mol^-1^.cm^-1^). HRMS (ESI-): [M-H]^-^ = 1310.6747 (calcd for C_88_H_88_N_5_O_6_: 1310.6740).

dye **3**. 230 mg of **9** (0.18 mmol, 1 eq.) was dissolved in 5 mL of anhydrous CH_2_Cl_2_, and the solution was added to 0.29 mL of DIEA (1.75 mmol, 10 eq.) and stirred for 1 hour at RT. Then, 0.33 mL of BF_3_.Et_2_O (2.63 mmol, 15 eq.) was added dropwise, and the solution was stirred for 15 h at room temperature. The reaction mixture was added to 20 mL of CH_2_Cl_2_ and washed with a saturated aqueous solution of NH_4_Cl (30 mL) and brine (30 mL). The organic layer was then dried over Na_2_SO_4_ and concentrated. Purification by flash chromatography on silica gel with CH_2_Cl_2_/petroleum ether as the eluent (3:7, R_f_ = 0.56) produced a dark-green solid product with 80 % yield (191 mg). ^1^H NMR (CDCl_3_, 500.10 MHz): δ 8.30-8.26 (m, 6H, CH_Ar_), 8.20 (d, ^4^J = 2 Hz, 2H, CH_Ar_), 8.17 (d, ^3^J = 9 Hz, 4H, CH_Ar_), 7.78 (d, ^3^J = 8 Hz, 2H, CH_Ar_), 7.73 (d, ^3^J = 8 Hz, 2H, CH_Ar_), 7.56-8.50 (m, 6H, CH_Ar_), 7.40 (d, ^3^J = 8 Hz, 2H, CH_Ar_), 7.34 (s, 2H, CH_Ar_), 7.08 (d, ^3^J = 9 Hz, 4H, CH_Ar_), 3.93 (s, 6H, OCH_3_), 2.00 (m, 8H, CH_2_), 1.14-1.01 (m, 24H, CH_2_), 0.77 (t, ^3^J = 7 Hz, 12H, CH_3_), 0.58 (m, 8H, CH_2_). ^13^C NMR (CDCl_3_, 125.75 MHz): δ 162.3 (C_quat_), 161.0 (C_quat_), 152.6 (C_quat_), 152.4 (C_quat_), 147.5 (C_quat_), 146.8 (C_quat_), 144.8 (C_quat_), 143.3 (C_quat_), 139.1 (C_quat_), 132.9 (t, J_C-F_ = 4 Hz, CH), 131.8 (C_quat_), 130.8 (CH), 130.8 (CH), 129.8 (CH), 128.3 (CH), 125.8 (CH), 123.9 (C_quat_), 123.6 (CH), 122.6 (C_quat_), 121.4 (CH), 120.3 (CH), 118.4 (CH), 113.7 (CH), 113.6 (C_quat_), 98.5 (C_quat_), 85.9 (C_quat_), 55.9 (C_quat_), 55.6 (OCH_3_), 40.1 (CH_2_), 31.6 (CH_2_), 29.7 (CH_2_), 23.9 (CH_2_), 22.7 (CH_2_), 14.1 (CH_3_). ^19^F NMR (CDCl_3_, 188.81 MHz): δ -131.1 (quad, J_F-B_ = 30 Hz). UV-Vis (CH_2_Cl_2_): λ_max_ = 762 nm (ε_max_ = 100000 L.mol^-1^.cm^-1^). HRMS (ESI+): [M+Na]^+^ = 1382.6677 (calcd for C_88_H_88_BF_2_N_5_NaO_6_: 1382.6701).

**2. Quantitative calculation of the switching charge.**

The percentage of the reduced dye in OFF state was determined from the following equation: R(%) = [Q / Q_Total_] x 100 (%), where Q is injected/ejected charge through the switching, and Q_Total_ is the total amount of charge that is required to one-electron reduce all the BDP dye in the device. Q was recoreded from the potentiostat [model CHI 624B (CH Instruments, Inc.)] during the electrochemical conversion. Q_Total_ was calculated by multiplying the concentration of the dye by the volume of the switching device, using the equation: Q_Total_ = F * C * V, where F is the Faraday constant (96485 C/mol), C is the concentration of the dye, and V is the volume of the switching device (16 mm * 20 mm * 0.3 mm). Because of the high diffusion coefficient, the R value for dye **4** showed the highest value among the dyes. We calculated the ratio of the reduced dye during the EF switching (R%) of the device in different electrolyte medium and under different applied potentials (Table S1 and Table S2), respectively. It was noteworthy that the R% was almost saturated after 50 s regardless of the switching potential. Whereas, with 10 s duration time, the high switching potential could result in fast reduction, as shown in Table S2. The highest value of dye **4** was found to be 40.5 % and 7.5 %, in each solvent, respectively.

**
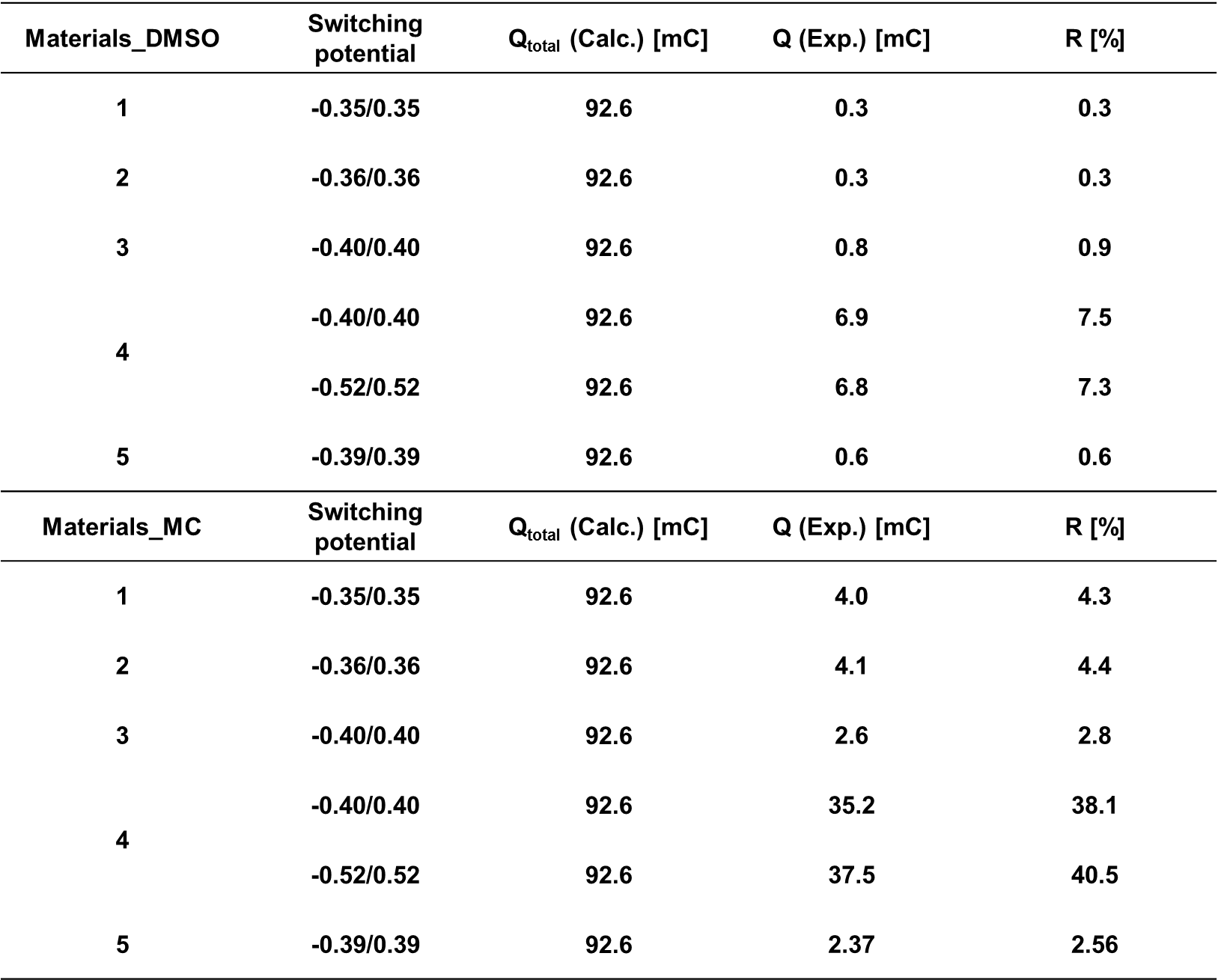
**

**Table S1.** Quantitative calculation of the switching charge for the aza-boron-dipyrromethenes dyes with (a) dichloromethane electrolyte and (b) DMSO electrolyte. Switching potentials were determined using the cyclic voltammetry redox peak.


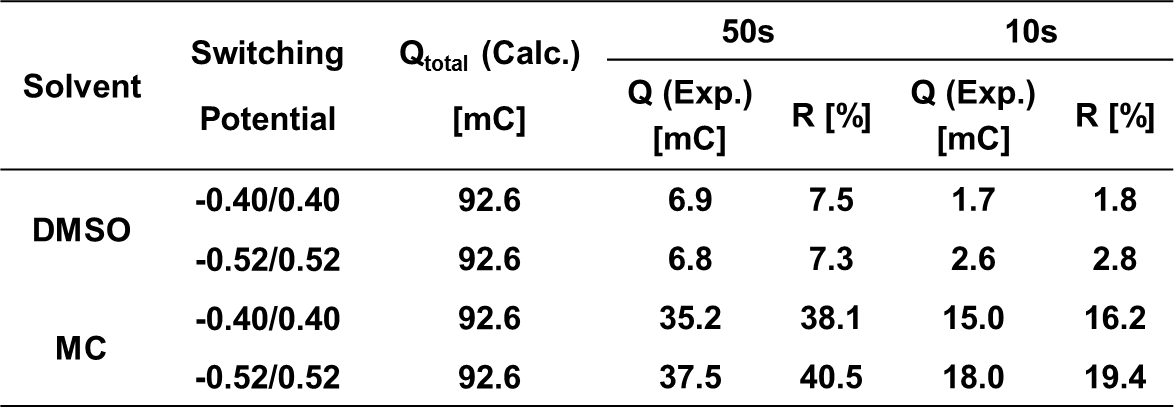


**Table S2.** Inject/eject charge of dye **4** were measured in different time conditions to show saturation effect.

**3. NIR electrochemical fluorescence imaging system**

As a detector, the conventional digital camera was used after removal of the inner band-pass filter to obtain NIR images. The NIR imaging system was designed to extract an NIR signal from the detectable light of the CCD. With a <720 nm cut-off, the CCD achieved the optical signal beyond 720 nm.


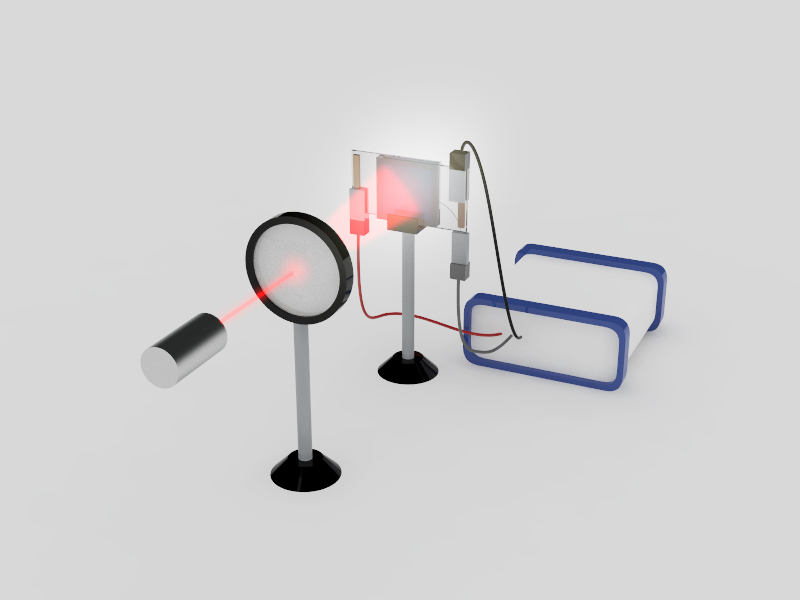


**Figure S1.** The schematic diagram of the electrofluorochromic switching system, which consists of the excitation source switching device used to obtain the NIR fluorescent images in Fig. 5-(b) and Fig. S11-(b)

**4. Optimized structure by density function theory (DFT) method using Gaussian 09 program**


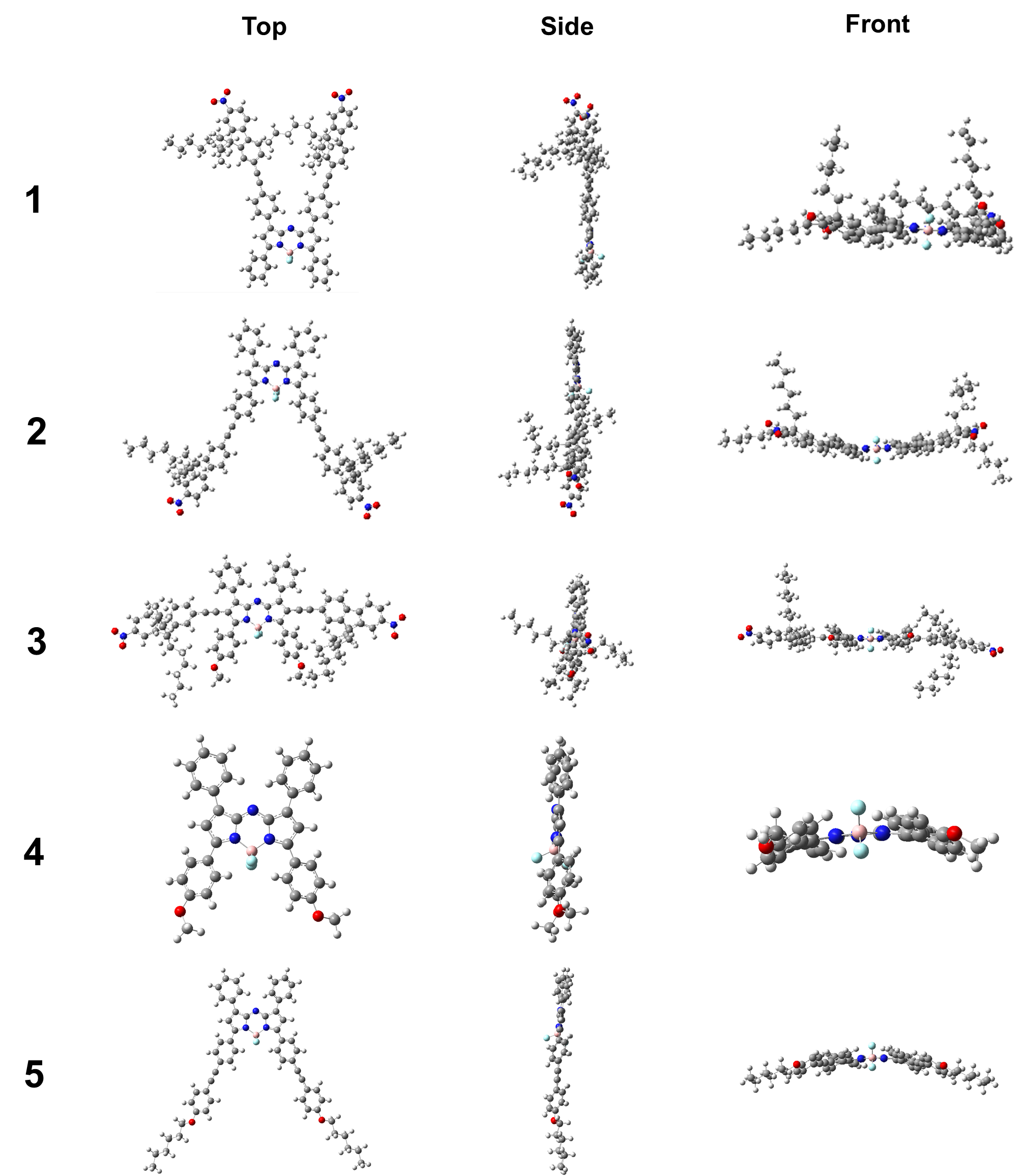


**Figure S2.** Optimized structure of aza-BDP dyes showed top, side, front views calculated by DFT method.


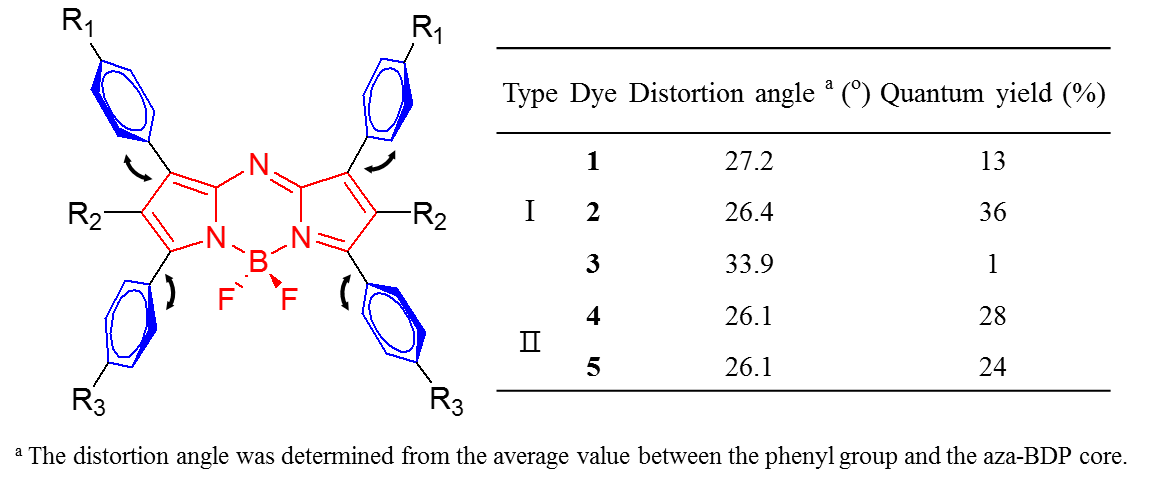


**Figure S3.** The distortion angle was calculated in the average value among four side phenyl rings from the optimized structure. The optimized structure was calculated by density function theory (DFT) method using Gaussian 09 program.

**5. Cyclic voltammetry of aza-boron-dipyrromethene dyes (one-electron oxidation process)**


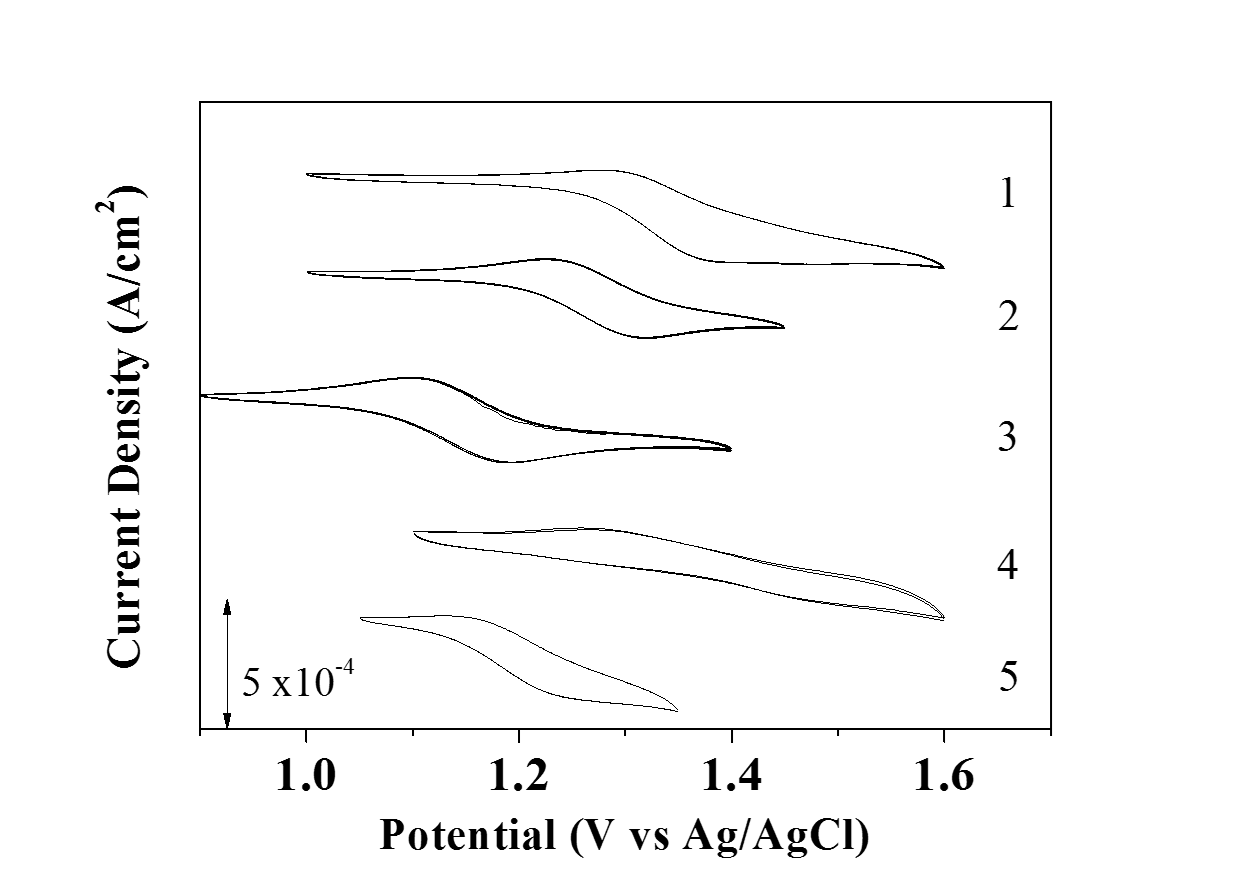


**Figure S4.** Cyclic voltammogram of the aza-boron-dipyrromethene dyes in an electrolyte containing TBAPF_6_ 0.2 M as a salt in dichloromethane. The dyes were measured in a solution state of 10^-3^ M concentration in the electrolyte, with a Ag/AgCl wire reference electrode, and Pt disk working electrode. (Scan rate = 100 mV/s) The potential range was determined to show the one-electron oxidation process.

**6. Complemental electrofluorochromic switching data of dyes**


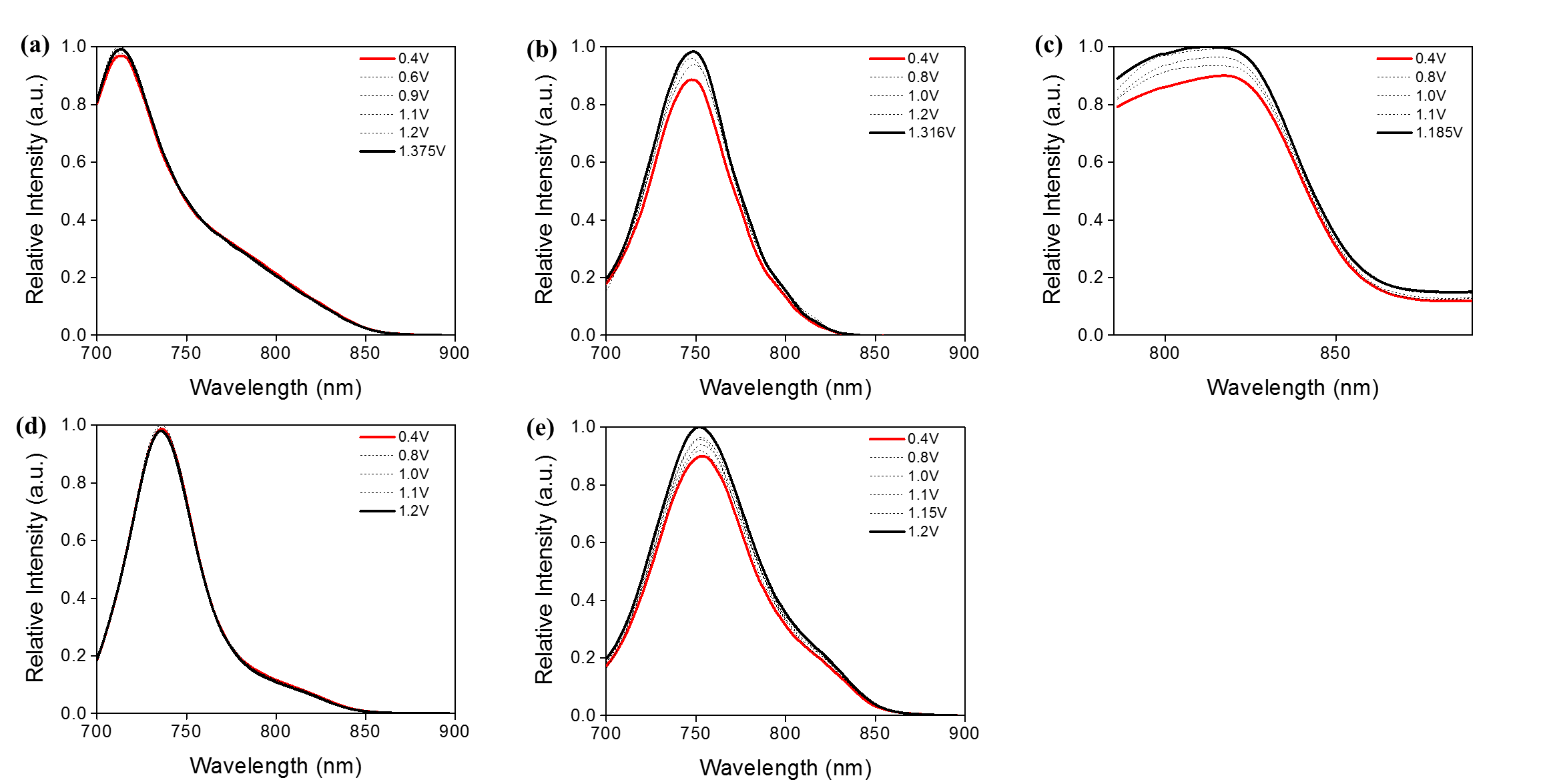


**Figure S5.** (a)-(e) represent the fluorescence changes of the **1**, **2**, **3**, **4** and **5** from neutral to oxidized states, respectively.


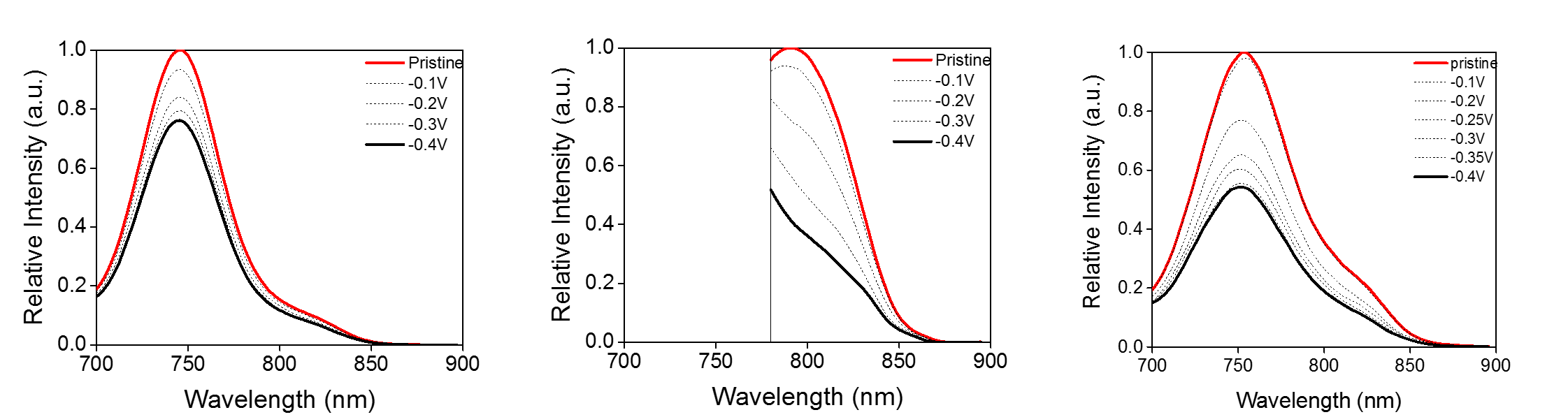


**Figure S8.** (a)-(c) represent the fluorescence changes of the **2**, **3** and **5**, respectively. With the dye **3**, the emission band was presented from 785 nm wavelength to avoid contribution from the strong excitation signal.

**
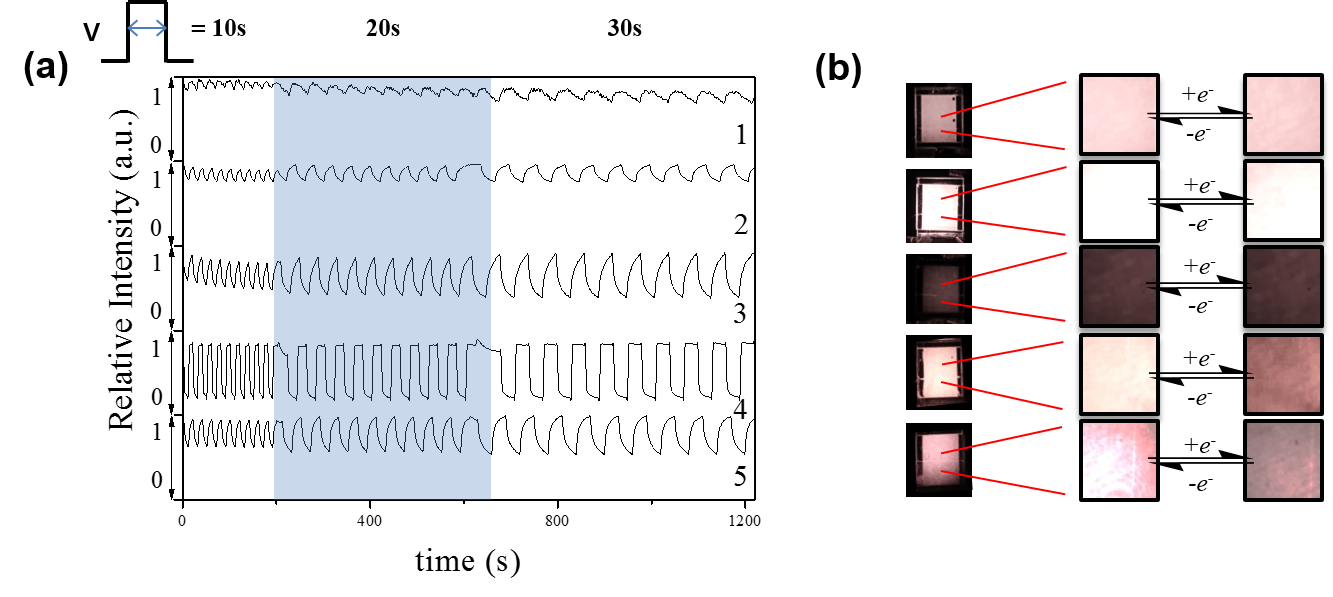
**

**Figure S11.** (a) Electrofluorochromic switching responses of aza-boron-dipyrromethenes with various step duration times of 10 s, 20 s, 30 s at each potential. (b) NIR images of electrofluorochromic switching devices applied by their switching potential to show ON/OFF NIR emission switching.

**7. Optimization of the electrofluorochromic working potential and electrofluorochromic switching contrast.**

**Figure S6.** Electrofluorochromic switching device containing dye **4** was examined with various switching potentials to find the optimum switching condition, with a 10 s step-duration time. The each experiment switching potentials were presented on the graph.

**
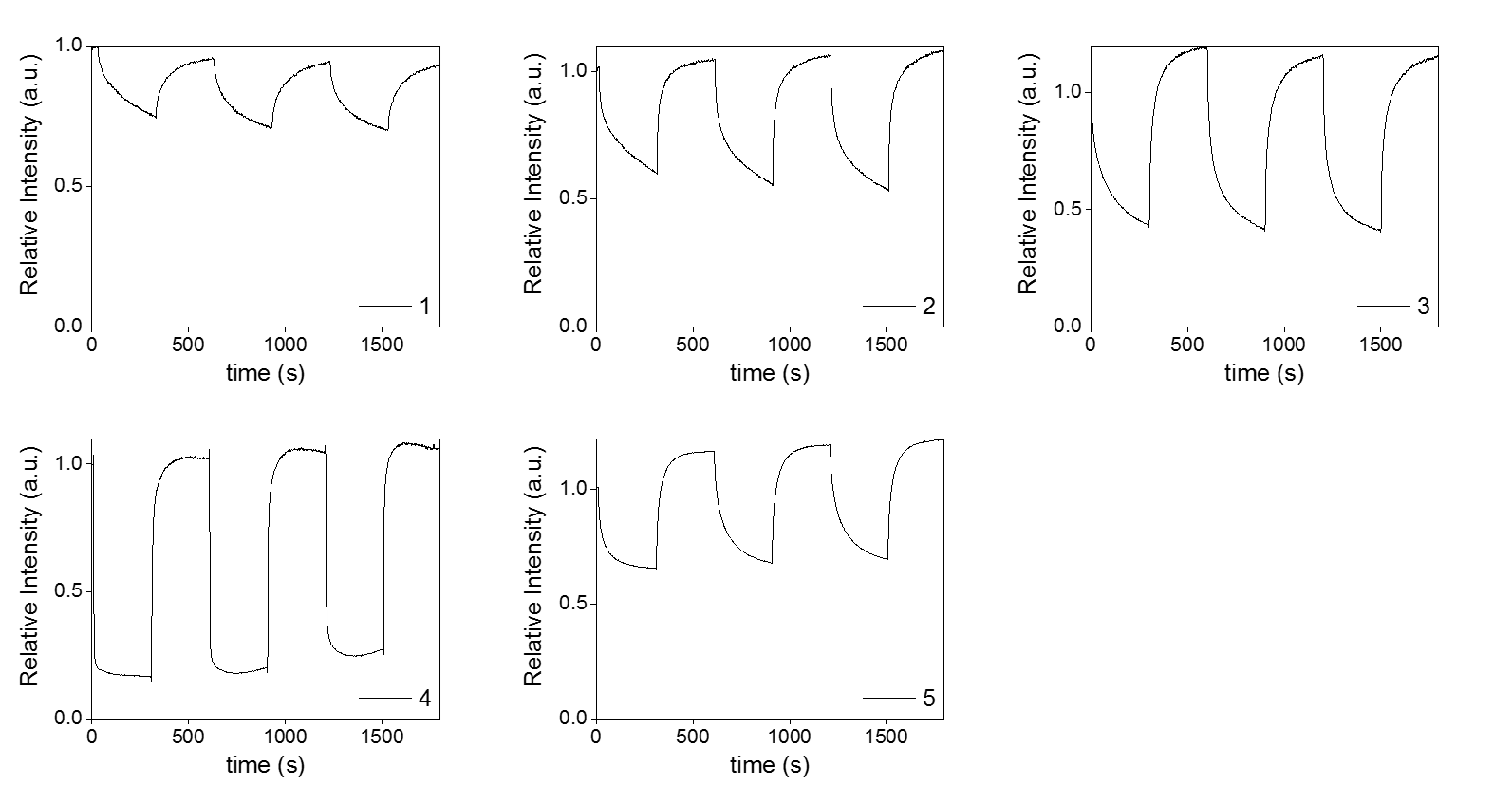
**

**Figure S10.** Electrofluorochromic switching graph for the aza-boron-dipyrromethenes dyes in 300 s duration times. Dye **4** showed the highest switching contrast with square-shaped response.

**8. Absorption changes of EF switching devices**

UV/Vis spectra of EF switching devices were obtained under different applied potentials. Upon applying the reduction and oxidation potentials, the absorption spectra of aza-BDPs were not significantly changed, except those of **4** and **5**, which showed visible change in the absorption upon oxidation because of the decomposition in the one electron oxidation process, as described above. This result supports that the fluorescence switching of aza-BDPs could be ascribed to the electrochemical conversion by one-electron reduction process, without the contribution from the electrochromic effects.


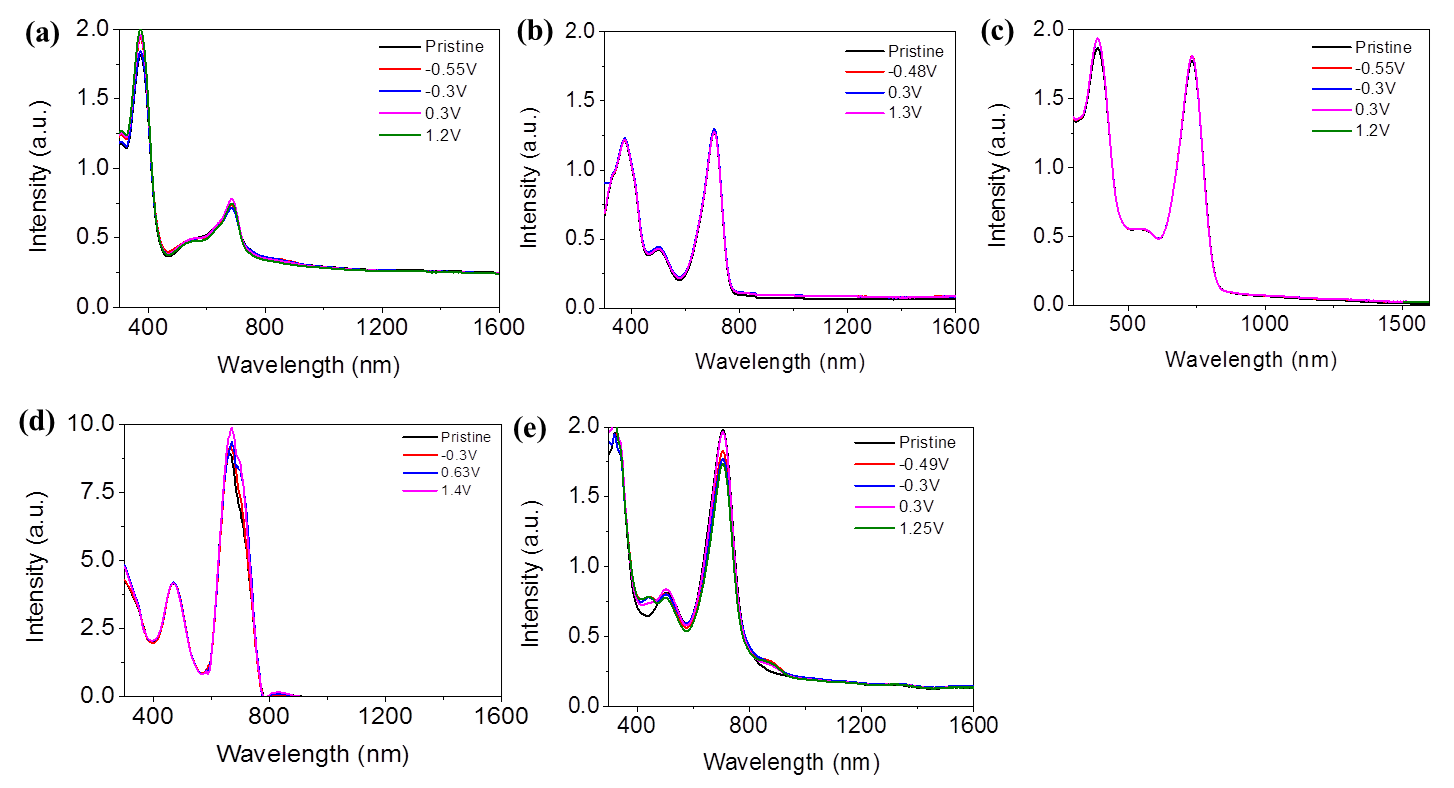


**Figure S7.** (a)-(e) represent the absorbance changes of the **1**, **2**, **3**, **4** and **5**, respectively. Absorbance was measured in three electrode system with applied potentials. A reference device was consist of an electrolyte containing Butvar B-98 and TBAPF_6_. We applied potentials from reduced to oxidized states to verify the absorbance changes by electrochemical process.

**9. Cyclability of the electrofluorochromic switching device containing aza-boron-dipyrromethenes.**

The cyclability of the EF switching device containing aza-BDP dyes was examined within the working potential range with a step-duration time of 10 s and 30 s. The fluorescence intensity was normalized by the intensity of the pristine state. The reversible switching continued over 1000 cycles with approximately less than 30 % of fluorescence contrast loss.

**
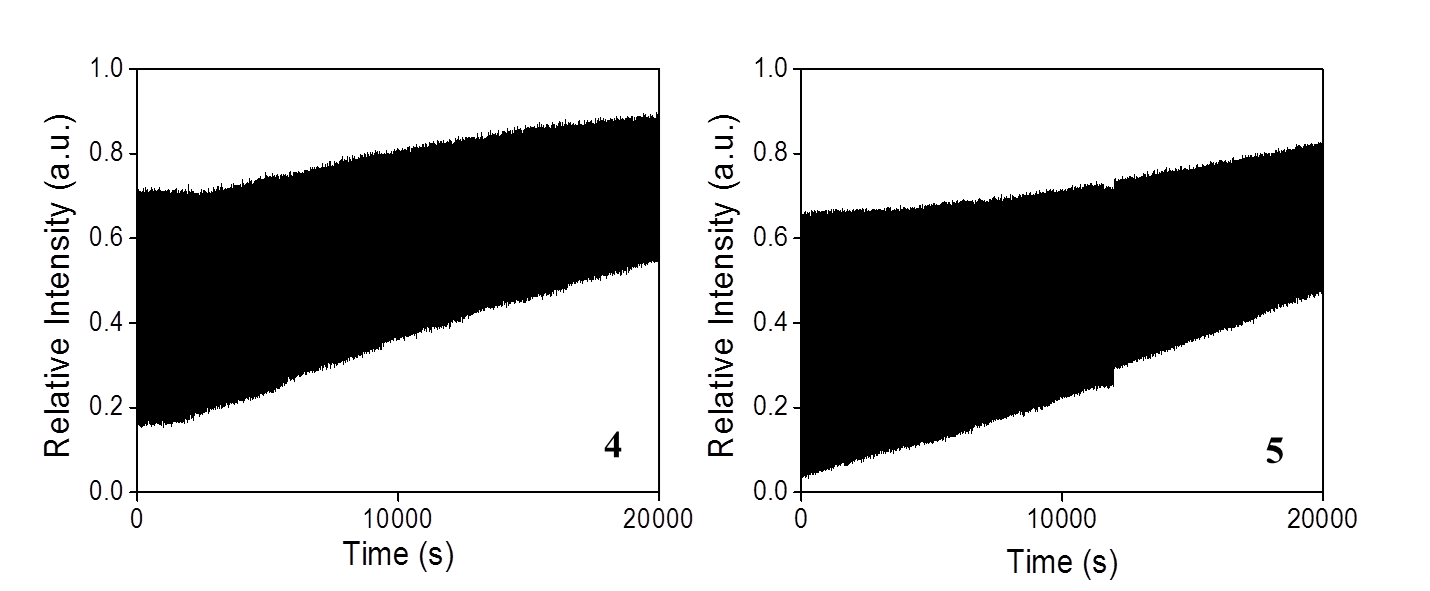
**

**Figure S9.** The cyclability test of the high-contrast electrofluorochromic device for dye **4** and **5** at 0.4 V with 10 s step-duration time from reduced to neutral states. λ_exc_ was determined as its absorption maximum, 686 nm. Cyclability was measured over 20000 s. The ON/OFF contrasts were maintained more than 1000 cycles, with ~ 20 % loss from the initial switching ON/OFF ratio.

**
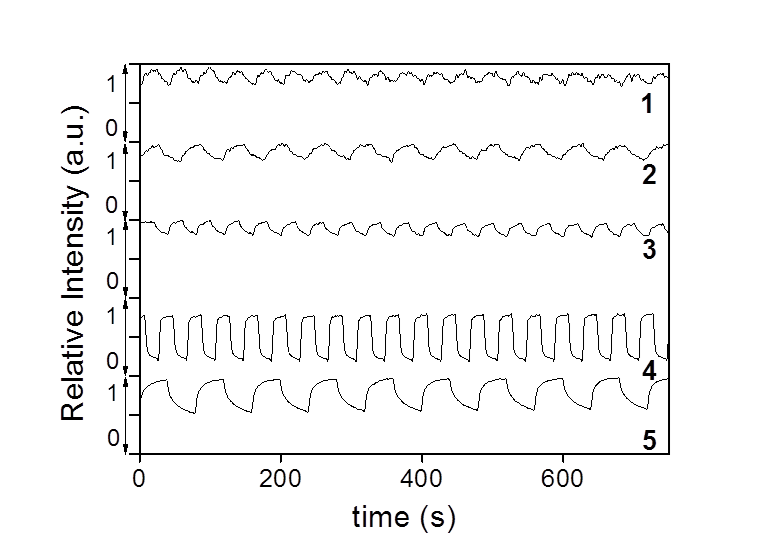
**

**Figure S13.** The fluorescence switching responses of the electrofluorochromic device containing dyes **1**, **2**, **3**, **4**, and **5** were measured for 10 min with 30 s of step-duration time at their working potentials. The applied potential was (0.35 V/- 0.35 V), (0.36 V/- 0.36 V), (0.4 V/- 0.4 V), (0.4 V/- 0.4 V), and (0.39 V/- 0.39 V) for dyes **1**, **2**, **3**, **4**, and **5**, respectively. λ_exc_ was determined as their absorption maximum.

**10. Calculation of diffusion coefficient (D)**

By the change of scan rate in cyclic voltammetry, the diffusion coefficient (D) was calculated according to the Randles-Sevcik equation as shown below:


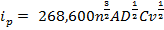
 , at 25 °C solution –eq2

where i_p_ is maximum current in amps, n is the number of electrons transferred in the redox event (usually 1), A is the electrode area in cm^2^, D is the diffusion coefficient in cm^2^/s, C is the concentration in mol/cm^3^, and v is the scan rate in V/s. By plotting the aniodic peak current obtained between - 0.3 V and - 0.5 V against v^1/2^ for every aza-BDP dyes, linear relationships were observed. The linear relationships indicate that the behavior of the electrochemical processes for the three-electrode device is mainly controlled by the diffusion of the dyes. The diffusion coefficient values of the dyes (D_f_) were determined from the slopes in Fig. S12. The D_f_ value of **4** is the highest value among the aza-BDP dyes. When the dyes were EF switched, D_f_ implies how fast it was quenched. Only dye **4** had a square EF switching graph among the dyes as shown in Fig. S10. The charge density value in Table S2 was strongly related to the D_f_ value.


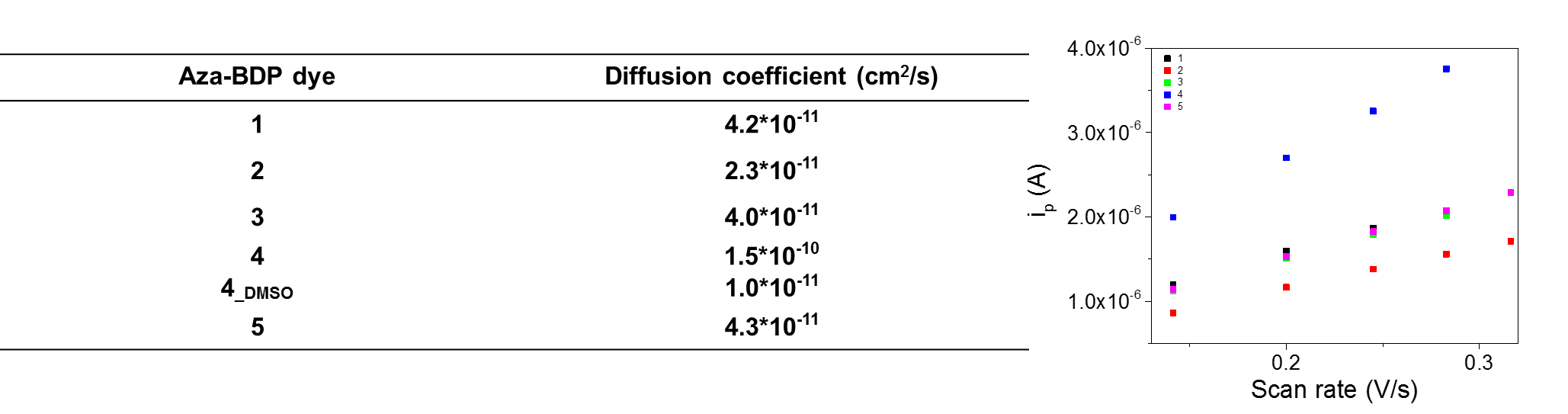
**Figure S12.** Calculation table of the diffusion coefficient (D_f_) by plotting the scan rate (V/s) versus the i_p_ (A), in MC solvent. Solvent effect of diffusion coefficient was tested in DMSO with dye **4**.

**11. Supporting Movies**

**Movie S1**. Electrofluorochromic switching movie of dye **4** with 10 s duration time from redox to neutral states. λ_exc_ was 684 nm. The switching potential was (0.5 V/-0.5 V).

**Movie S2**. Electrofluorochromic switching movie of dye **4** with 50 s duration time from redox to neutral states. λ_exc_ was 684 nm. The switching potential was (0.5 V/-0.5 V).

**12. Summary of electrofluorochromic materials**

Previously reported EF materials are summarized in Table S4, with their EF performances, such as contrast (= ON/OFF ratio), wavelength, cyclability, response time and potential ranges. Among the EF materials, the aza-BDPs showed compatible ON/OFF ratio, response time and potential ranges. On the other hands, compare to another NIR emissive EF material PM1 [Chem. Sci, 2014, 5, 1538] aza-BDP showed dramatically enhanced cyclability (> 1000 cycles).

**
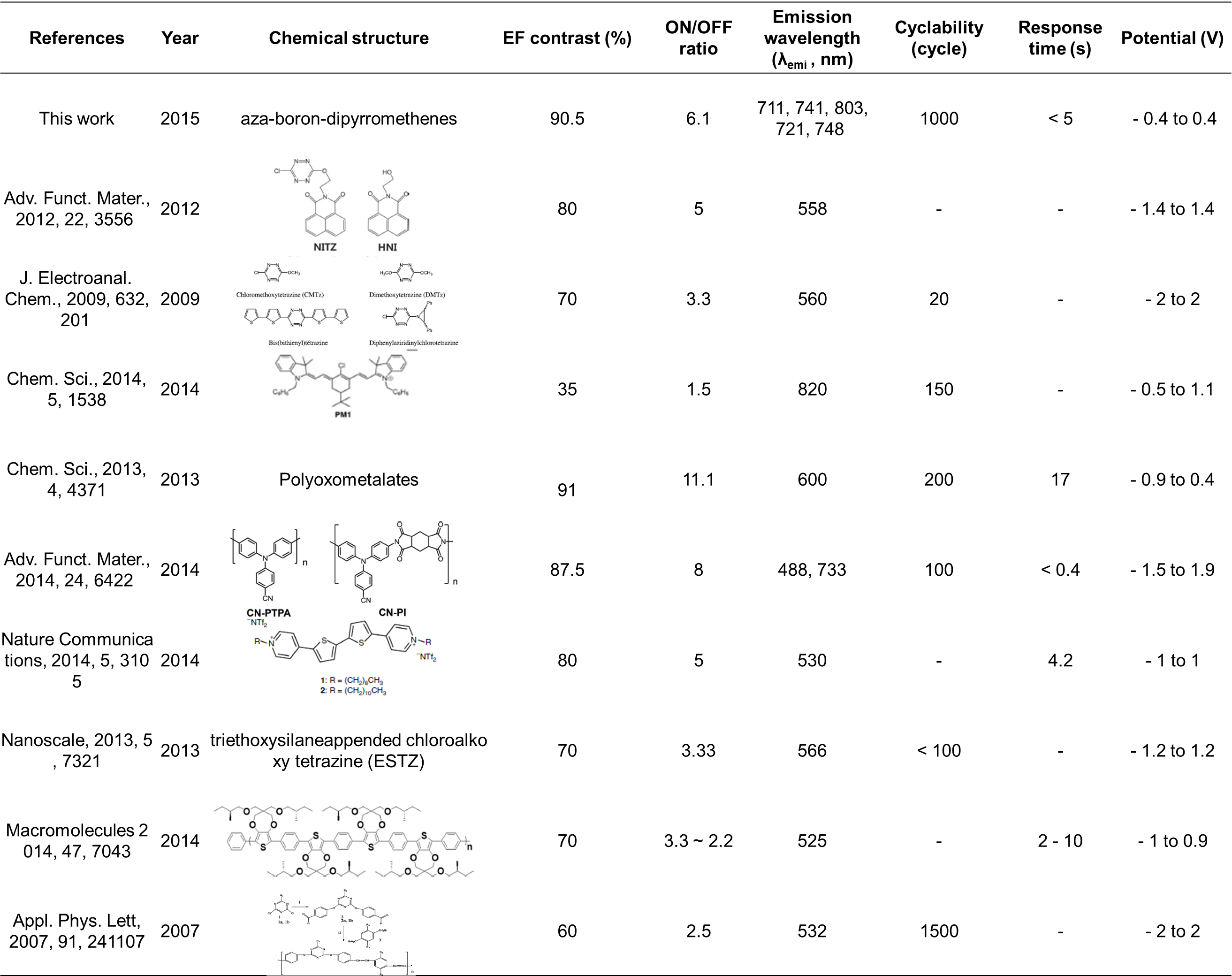
**

**Table S3**. EF performances of representative EF materials.
